# No genetic association between iron deficiency anemia and ischemic stroke and its subtypes: a bidirectional two-sample Mendelian randomization study

**DOI:** 10.3389/fneur.2024.1408758

**Published:** 2024-08-19

**Authors:** Xingyu Chen, Aiping Li, Wensheng Zhou, Liping Yao

**Affiliations:** ^1^Department of Neurology, Hunan Provincial People’s Hospital, The First Affiliated Hospital of Hunan Normal University, Changsha, China; ^2^Department of Neurology, The Third Hospital of Changsha, Changsha, China

**Keywords:** iron deficiency anemia, ischemic stroke, small vessel stroke, cardioembolic stroke, large artery stroke, Mendelian randomization

## Abstract

**Background:**

Observational researches have suggested a connection between iron deficiency anemia (IDA) and an increased likelihood of ischemic stroke (IS), yet establishing causality is challenging owing to the inherent limitations of such studies, including their vulnerability to confounding factors and the potential for reverse causation. This study employs a bidirectional two-sample Mendelian randomization (MR) approach to assess the causal linkage between IDA and IS and its subtypes.

**Methods:**

Identifiable single nucleotide polymorphisms (SNPs) with significant links to either IDA or IS and its subtypes were employed as instrumental variables (IVs). The relationship between IDA and any IS, small vessel stroke (SVS), cardioembolic stroke (CES), and large artery stroke (LAS), was quantified using the inverse variance weighted (IVW) method. Complementary analyses utilizing MR-Egger and weighted median methods further supplemented the IVW findings. Moreover, the leave-one-out analysis, MR-Egger intercept test, MR-PRESSO global test, and Cochrane’s Q test were conducted for sensitivity analyses.

**Results:**

This study revealed no correlation between IDA and any IS (IVW method: OR [95% CI] = 0.977 [0.863–1.106]; *p* = 0.716), LAS (OR [95% CI] = 1.158 [0.771–1.740]; *p* = 0.479), CES (OR [95% CI] = 1.065 [0.882–1.285]; *p* = 0.512), or SVS (OR [95% CI] = 1.138 [0.865–1.498]; *p* = 0.357). Conducting a reverse MR analysis, it was determined that there is no causal connection between any IS, LAS, CES, SVS, and IDA (all *p* > 0.05). Sensitivity analysis indicated that heterogeneity was not significant and no evidence of horizontal pleiotropy was detected.

**Conclusion:**

This MR study suggested no causal effect of IDA on IS, LAS, CES, and SVS. Through reverse MR analyses, it was determined that IS and its subtypes did not exert a causal impact on IDA.

## 1 Introduction

Stroke represents a major global health challenge and a leading cause of mortality, affecting approximately 80 million people worldwide and representing the second greatest burden of disease globally (1). Of all stroke types, ischemic stroke (IS) accounts for approximately 87% (2), with the major causative categories being small vessel stroke (SVS), cardioembolic stroke (CES), and large artery stroke (LAS) (3). Characterized by high rates of mortality and disability, IS is a principal contributor to human disability and death, causing neurological impairments such as hemiplegia and aphasia that profoundly diminish individuals’ quality of life (4). Despite substantial progress in managing traditional contributing factors-including diabetes mellitus, hyperlipidemia, and high blood pressure-through medical interventions, the incidence of IS continues to be a paramount public health issue worldwide (5). Given complex etiology of IS and the involvement of multiple risk factors (6), the early detection and management of these risks are critical (7).

Anemia, designated as the fifth cardinal cardiovascular risk factor (8), exhibits a global prevalence of 32.9%, making it one of the most widespread medical conditions (9). It is typified by diminished levels of hemoglobin or hematocrit, leading to reduced oxygen transport capability of the red blood cells, with potential systemic effects (10). Among the various causes of anemia, iron deficiency anemia (IDA) emerges as a predominant factor worldwide (11). IDA has been documented as an infrequent etiological factor for IS. In certain case reports, instances of thrombosis in the carotid arteries have been documented, with aortic thrombosis being a rarer occurrence, in patients suffering from IDA (12, 13). Findings from case–control investigations suggested a notable correlation between IDA and the increased incidence of stroke, specifically IS (14, 15). An observational study revealed that the incidence of IS in patients with IDA was 24.4% (782/3199) (14). Several mechanisms may elucidate the potential link between IDA and increased IS risk. The reduction in hemoglobin levels associated with IDA compromises oxygen transport, potentially leading to tissue hypoxia, including in cerebral regions, thereby elevating stroke risk (16). Additionally, the body’s compensatory response to anemia may involve enhanced erythropoiesis, resulting in increased red blood cell production and higher blood viscosity, which could contribute to thrombotic events (17). However, observational research, while indicating a relationship of IDA and ischemic IS risk, may be subject to biases from potential confounding factors and reverse causality. This underscores the limitations of these studies in conclusively establishing a causal association between IDA and IS risk.

Mendelian randomization (MR) serves as a statistical approach leveraging genetic variations as instrumental variables (IVs) for elucidating causative associations of exposure factors and outcomes (18). This model leverages the random allocation of genetic variants at conception, mitigating the influence of environmental confounders and the risk of reverse causation (19). Consequently, MR offers a robust alternative to traditional epidemiologic approaches (20). To date, the potential causal link between IDA and IS, along with its subtypes, has not been investigated through MR analysis. This study aimed to explore the causal relationship between IDA and IS and its subtype risk based on large-sample genome-wide association study (GWAS) databases through a bidirectional two-sample MR analysis.

## 2 Methods

### 2.1 Study design

**Figure 1:**
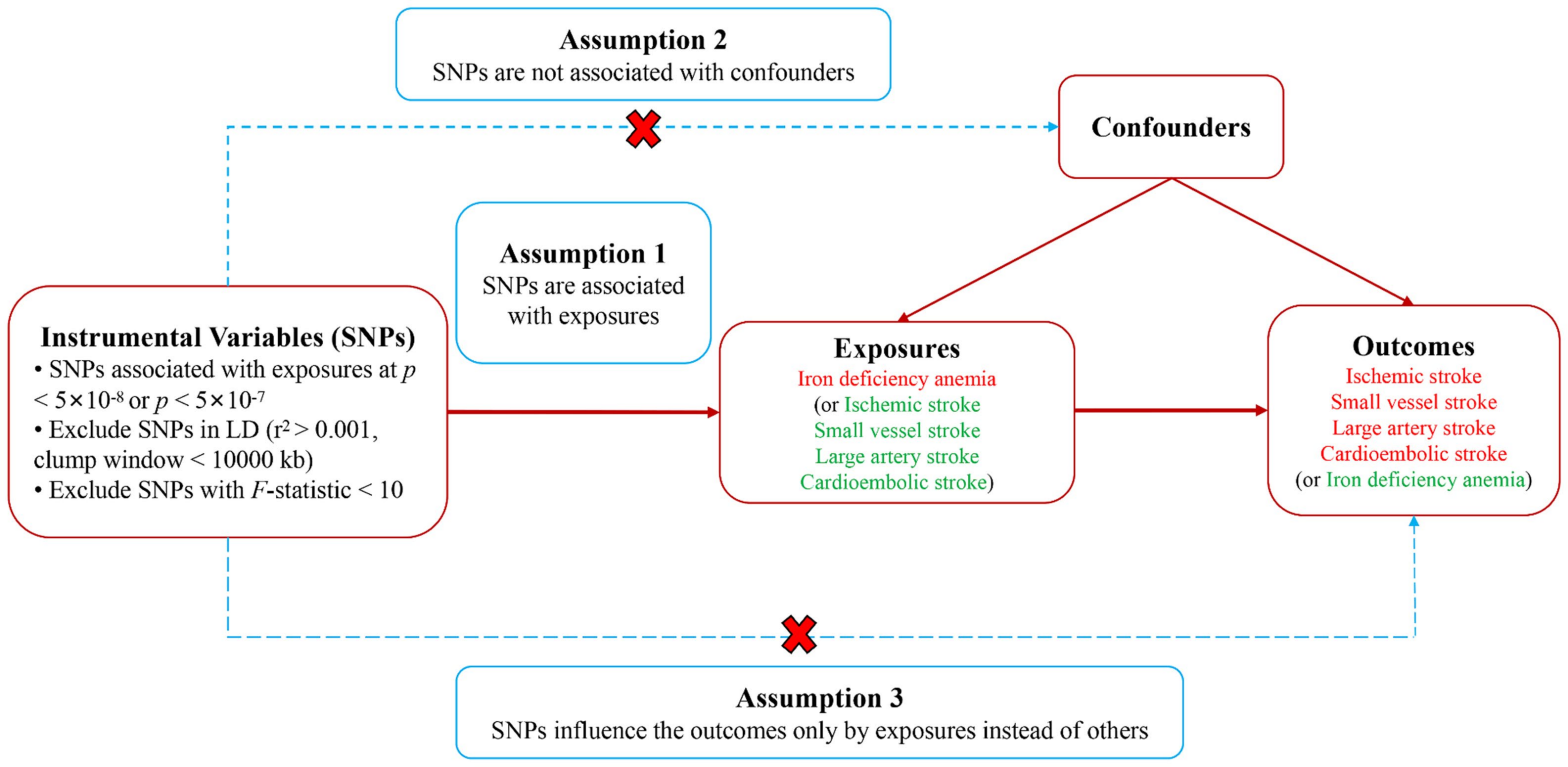
Study design diagram and three assumptions of Mendelian randomization. SNPs, single nucleotide polymorphisms.

In this research, we used a two-sample bidirectional MR approach to thoroughly evaluate the two-way causative link between IDA and IS and its subtypes. The research employed IVs, derived from genetic variances, particularly single nucleotide polymorphisms (SNPs), which show strong associations with IDA or IS and its subtypes. For the MR analysis to be deemed credible, it was imperative to satisfy three essential conditions: (1) A strong association existed between the IVs and exposure variables; (2) The selected SNPs had no links to any underlying confounders; (3) The IVs exclusively influenced the outcomes via the exposure mechanism (21). A brief overview of the bidirectional MR analysis was presented in Figure 1. Our methodology adhered to the guidelines outlined in the STROBE-MR for documenting our findings (22). Given that the data for our analysis were secondary data sourced from an existing study with prior ethical approval, the necessity for additional ethics approval and informed consent was circumvented in this case.

### 2.2 Data sources

Data from GWAS pertaining to IDA and SNPs were acquired from the FinnGen consortium,^1^ encompassing publicly accessible summary statistics for IDA and 21,306,290 SNPs from 408,837 European descent individuals. IS-related information was derived from the MEGASTROKE consortium’s European cohort (23, 24), involving European ancestry participants. The principal endpoint analyzed was IS, contrasting 34,217 cases and 406,111 control subjects. Distinct subcategories of IS explored included LAS, with an analysis of 6,399 cases; CES, involving 7,193 cases; and SVS, assessing 5,386 cases, against a control cohort of 1,234,808 for LAS, 192,662 for SVS and 406,111 for CES. Details of the GWAS are encapsulated in Table 1.

**Table 1:** Detailed information of the GWAS included in Mendelian randomization analysis.

Exposures/Outcomes	Consortium	Cases/Controls	nSNP	Ethnicity	PMID	Data source	Population	Year
Iron deficiency anemia	FinnGen	15,153/393,684	21,306,290	European	-	finngen_R10_D3_ ANAEMIA_IRONDEF	-	2023
Ischemic stroke	MEGASTROKE	34,217/406,111	8,338,157	European	29,531,354	GWAS catalog	Adults	2018
Large artery stroke	MEGASTROKE	6,399/1,234,808	5,774,938	European	36,180,795	GWAS catalog	Adults	2022
Cardioembolic stroke	MEGASTROKE	7,193/406,111	8,303,699	European	29,531,354	GWAS catalog	Adults	2018
Small vessel stroke	MEGASTROKE	5,386/192,662	8,311,897	European	29,531,354	GWAS catalog	Adults	2018

### 2.3 Instrumental variable selection

In the selection process for genetic variants related to exposures and outcomes, a standardized criterion was employed. Acknowledging the potential limitation in the number of SNPs reaching comprehensive genome-wide significance, we moderated the significance threshold to a *p*-value of less than 5 × 10^−7^ for LAS and SVS. Independent IVs were distinguished through a process of linkage disequilibrium (LD) clumping, applying an r^2^ threshold at 0.001 with a clumping distance of 10,000 kb, based on the LD reference derived from the 1,000 Genomes Project. Variants presenting the lowest *p*-values were selected as independent instruments (25). Further, SNPs demonstrating significant associations with the exposure variables were evaluated using the *F*-statistic, identifying strong IVs by the criterion *F* > 10 (26). The GWAS catalog^2^ was then used to assess potential correlations between selected SNPs and confounding factors. SNPs associated with exposures and directly linked to outcomes (*p* < 5 × 10^−8^) were excluded. Finally, the gathered data from exposure and outcome databases were merged, ensuring the elimination of palindromic sequences to maintain uniformity in effector alleles.

### 2.4 MR analysis

The primary method for estimating the causal effect between IDA and IS was the inverse variance weighted (IVW) approach, which derives weighted summaries based on the inverse of the variance, presuming the validity of all instrumental variables. As a pivotal method within MR research, IVW synthesizes Wald ratios from individual SNPs to generate an overall causal estimate (27). To assess the robustness of the results and examine the presence of pleiotropy, additional analyses were conducted using the MR-Egger regression and weighted median method. The MR-Egger method is capable of identifying pleiotropic effects via its intercept, offering adjustments for pleiotropy in its estimates, albeit with reduced statistical power (28). The weighted median method integrates data across numerous genetic variants, providing a consolidated causal estimate (29). The findings reached statistical significance with the *p*-value below 0.0125 (0.05/4) following the application of the Bonferroni correction for multiple tests. A *p*-value within the range of 0.0125 to 0.05 are considered indicative of potential statistical significance.

### 2.5 Sensitivity analysis

To assess the heterogeneity across individual genetic variance estimates, Cochran’s Q test was employed. A *p*-value from Cochran’s Q test less than 0.05 prompted the adoption of the random effects model for subsequent MR analysis, while a higher *p*-value warranted the use of a fixed-effects model (30). Assessment of horizontal pleiotropy relied on the Egger intercept, considering the absence of pleiotropy when *p* > 0.05 (28). To identify and mitigate the impact of outliers on causal inference, the MR pleiotropy residual sum and outlier (MR-PRESSO) approach was employed (31). The leave-one-out strategy was applied to identify IVs potentially impacting causal effect estimates, sequentially excluding each SNP to determine the meta-effect of the remaining SNPs. Visual representation of our findings was achieved through scatter and forest plots, elucidating the linkage between IDA and IS. Funnel plot analysis was conducted to verify the results’ stability. All statistical analyses were performed using R software (v4.3.1) with the TwoSampleMR and MRPRESSO packages (v0.5.7).

## 3 Results

### 3.1 Selection of instrumental variables

Using IDA as the exposure variable, we screened 7 SNPs as IVs, none of which were excluded because of their non-significant association with confounding variables. Consequently, 7 SNPs were used as IVs for causal effect analysis of IDA on IS, LAS, CES, and SVS. In the scenario where IDA served as the outcome variable, our research identified a total of 29 SNPs, segmented as 10 associated with IS, 9 with LAS, 4 with CES, and 6 with SVS. Among them, rs3184504 was excluded due to its significant correlation with IS as well as red blood cell count and hemoglobin levels. We also eliminated any SNPs that were palindromic and held a moderate allele frequency. The robustness of our instruments was supported by *F-*statistics exceeding 10 for all included SNPs, suggesting no weak instrument bias. Details about these SNPs was provided in Supplementary Tables S1–S13.

### 3.2 Causal effects of IDA on IS and its subtypes

**Figure 2:**
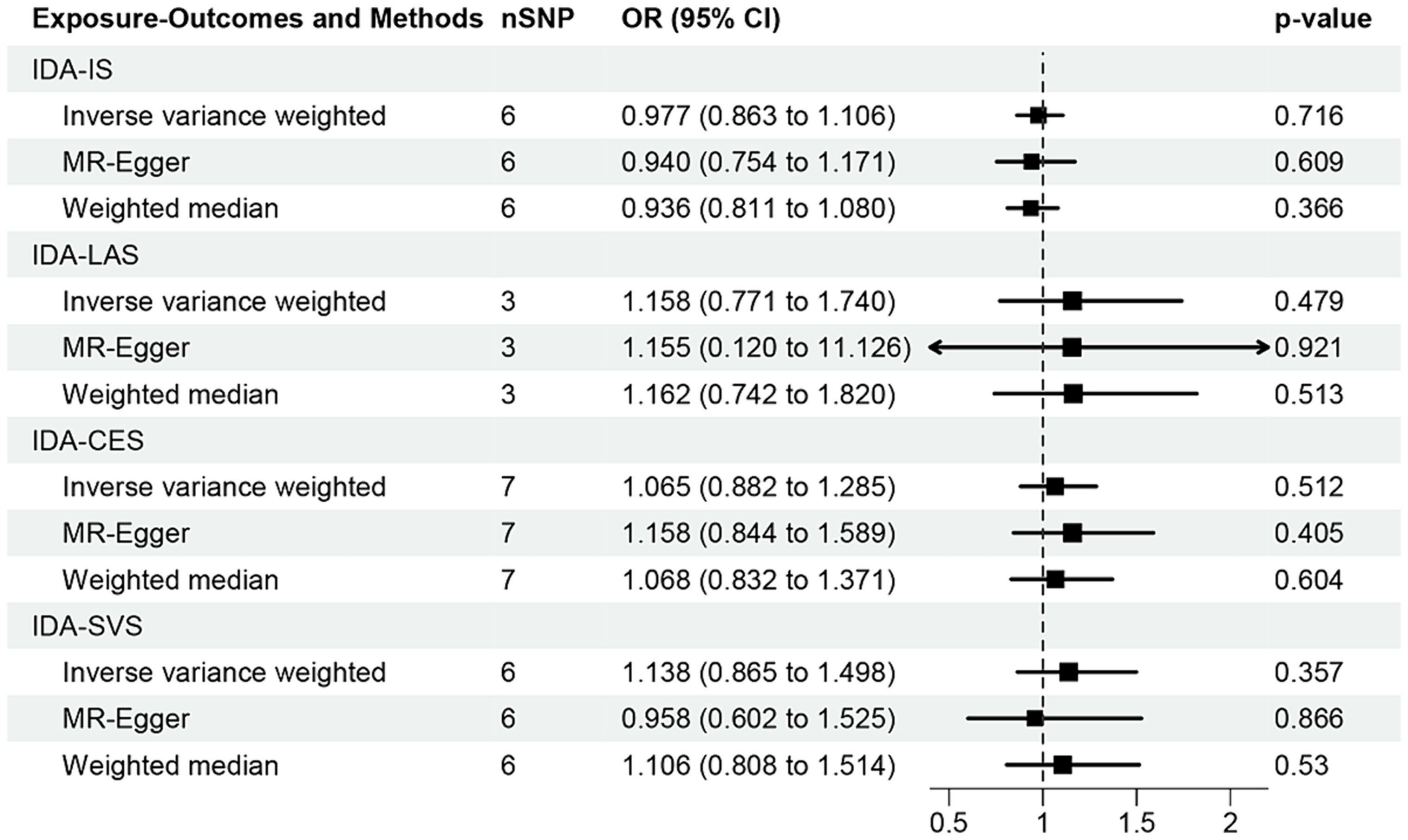
MR analysis of the causal effect of iron deficiency anemia on ischemic stroke and its subtypes. IDA, iron deficiency anemia; IS, ischemic stroke; LAS, large artery stroke; CES, cardioembolic stroke; SVS, small vessel stroke.

**Figure 3:**
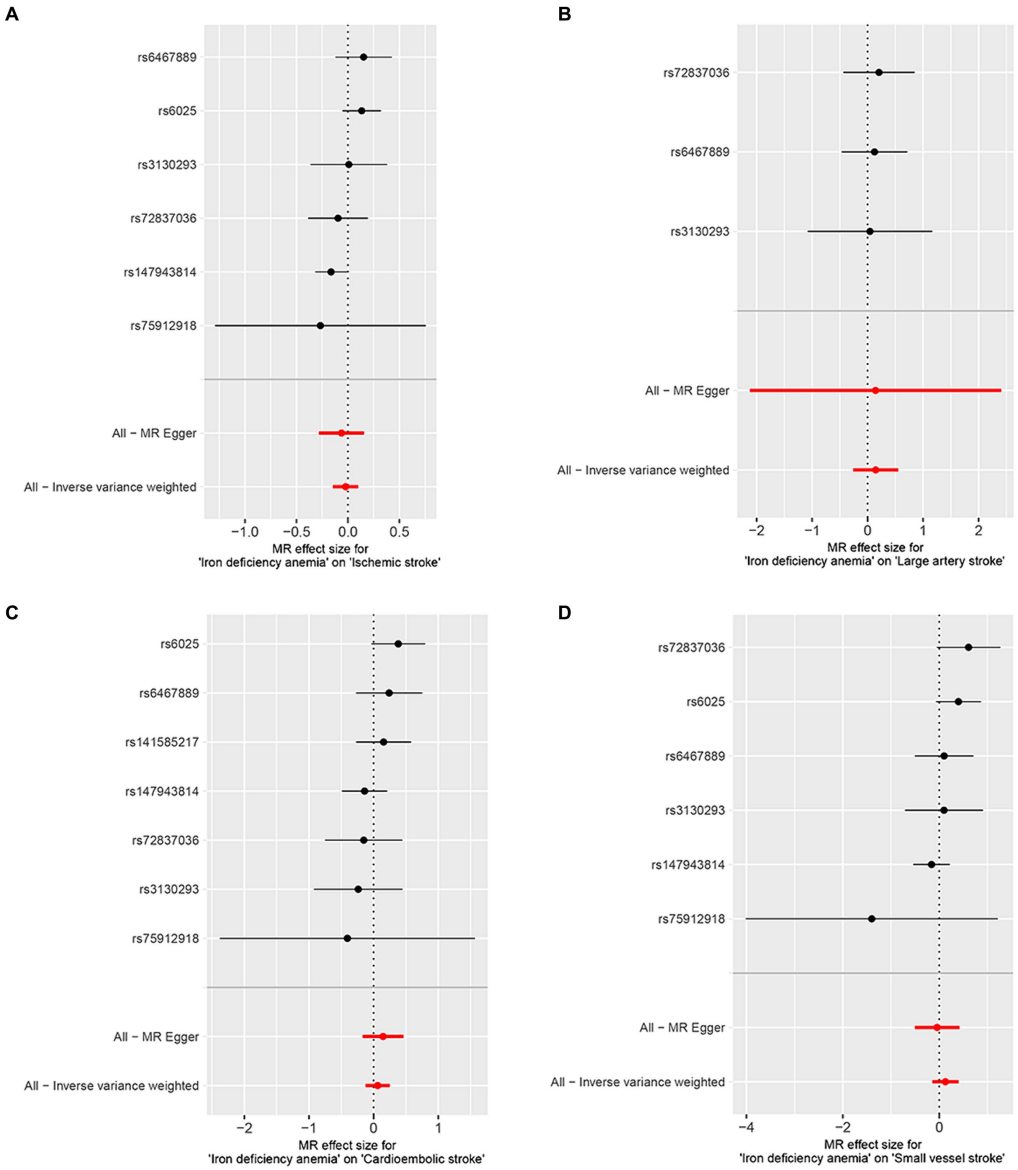
Forest plots of iron deficiency anemia on ischemic stroke and its subtypes. **(A)** Ischemic stroke; **(B)** large artery stroke; **(C)** cardioembolic stroke; **(D)** small vessel stroke.

**Figure 4:**
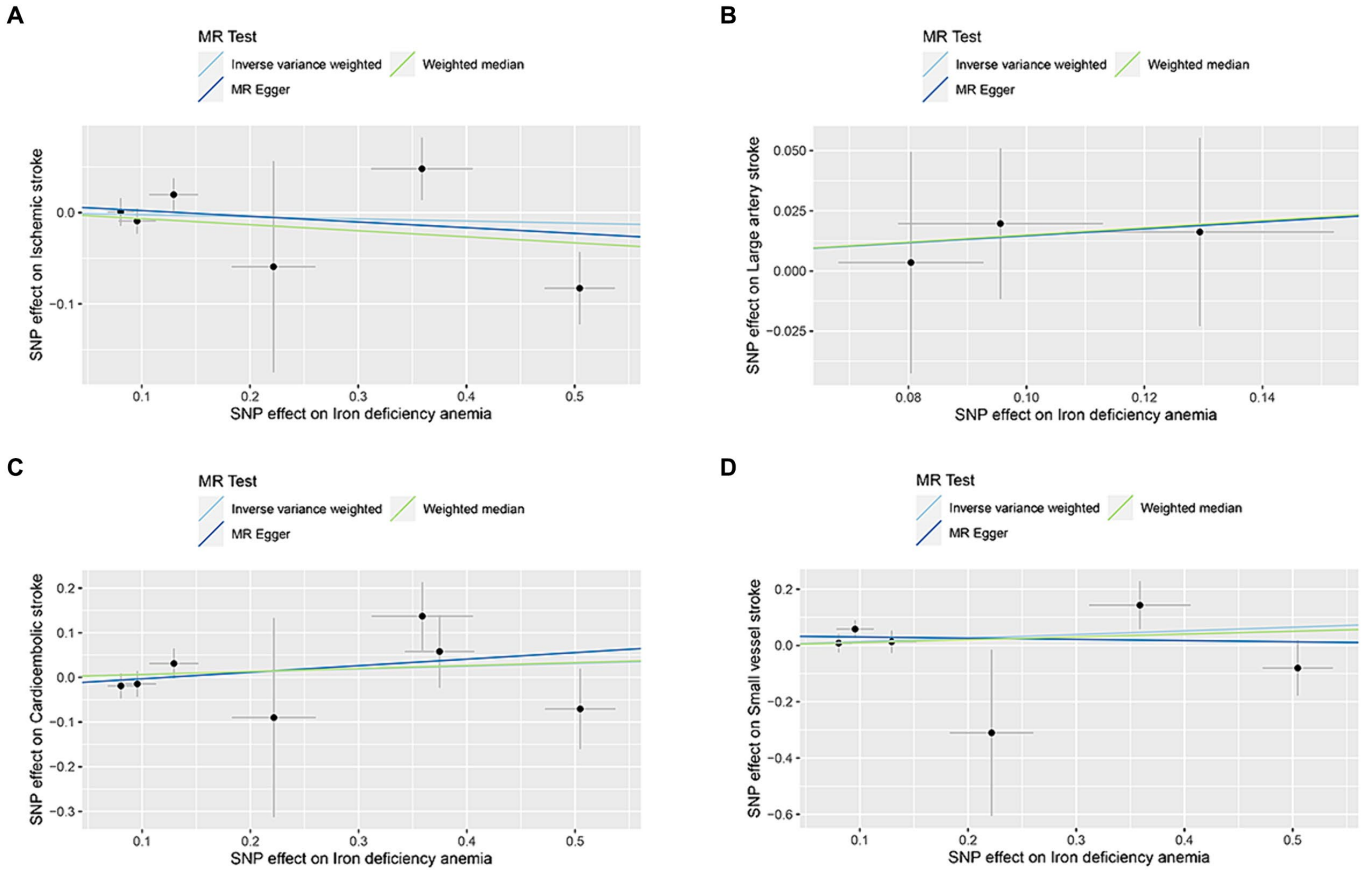
MR analysis scatter plots of iron deficiency anemia on ischemic stroke and its subtypes. **(A)** Ischemic stroke; **(B)** large artery stroke; **(C)** cardioembolic stroke; **(D)** small vessel stroke.

The correlation of IDA with IS and its various types was depicted in Figure 2. Utilizing the IVW approach, we observed no causal relationships between IDA with the incidence of IS (OR [95% CI] = 0.977 [0.863–1.106]; *p* = 0.716), LAS (OR [95% CI] = 1.158 [0.771–1.740]; *p* = 0.479), CES (OR [95% CI] = 1.065 [0.882–1.285]; *p* = 0.512), and SVS (OR [95% CI] = 1.138 [0.865–1.498]; *p* = 0.357). This observation was supported by both MR-Egger and weighted median analyses, with both of them showing no statistical significance (all *p* > 0.05). Forest plots in Figure 3 presented the estimates of causal effects between IDA and IS and its subtypes. Additionally, Figure 4 displayed scatter plots with MR intercepts approaching zero, suggesting a minimal presence of horizontal pleiotropy across the analyses.

### 3.3 Causal effects of IS and its subtypes on IDA

**Figure 5:**
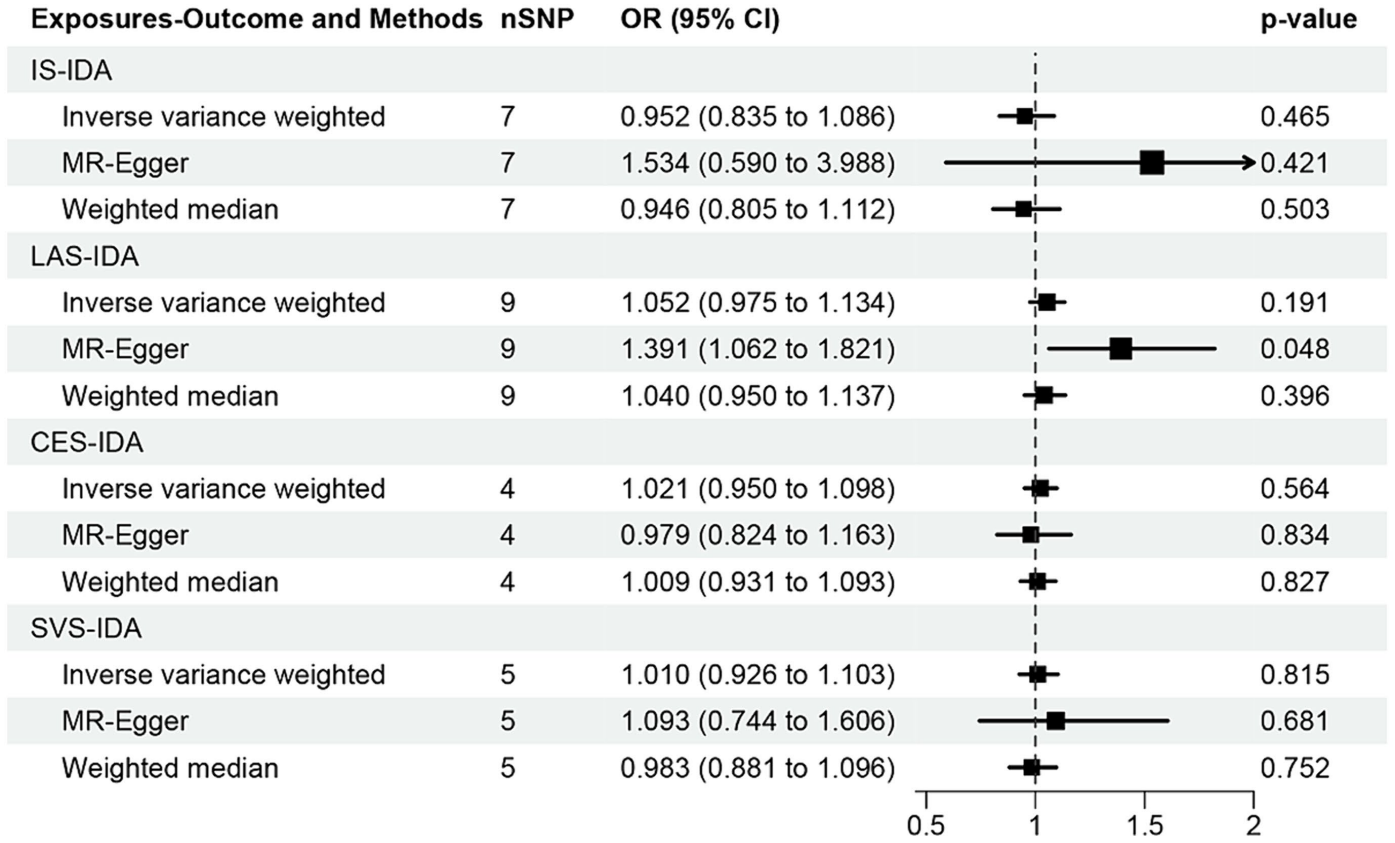
MR analysis of the causal effect of ischemic stroke and its subtypes on iron deficiency anemia. IS, ischemic stroke; IDA, iron deficiency anemia; LAS, large artery stroke; CES, cardioembolic stroke; SVS, small vessel stroke.

In the analysis conducted through the IVW approach, our study found no causal relationships of IS (OR [95% CI] = 0.952 [0.835–1.086]; *p* = 0.465), LAS (OR [95% CI] = 1.052 [0.975–1.134]; *p* = 0.191), CES (OR [95% CI] = 1.021 [0.950–1.098]; *p* = 0.564), and SVS (OR [95% CI] = 1.010 [0.926–1.103]; *p* = 0.815) with IDA (Figure 5). However, the MR-Egger method suggested that LAS may potentially increase the risk of IDA (OR [95% CI] = 1.391 [1.062–1.821]; *p* = 0.048). Additionally, no causal association between IS, CES, SVS, and IDA was found through MR-Egger and weighted median methods (all *p* > 0.05). The forest plots and scatter plots were shown in Supplementary Figures S1, S2.

### 3.4 Sensitivity analyses

The MR sensitivity analysis results are presented in Table 2. Upon conducting a heterogeneity test, all *p*-values derived from Cochrane’s Q statistics were found to be greater than 0.05, suggesting a lack of heterogeneity among the SNPs. Additionally, the MR-Egger regression intercept, used to assess horizontal pleiotropy, did not provide substantial evidence for the presence of pleiotropy. This lack of significant horizontal pleiotropy outliers was also corroborated by the MR-PRESSO results. The leave-one-out analysis did not indicate any significant influence of individual SNPs on the overall findings. The detailed outcomes of the leave-one-out analysis can be found in Supplementary Figures S3, S4. The funnel plots, depicted in Supplementary Figures S5, S6, did not reveal any significant bias, further corroborating the robustness of our results.

**Table 2:** Sensitivity analysis of the MR analysis results of iron deficiency anemia and ischemic stroke and its subtypes.

Exposures	Outcomes	Heterogeneity test		Pleiotropy test		MR-PRESSO
		Cochran’s Q Test	*p*	Egger Intercept	*p*	Global Test (*p*)
Iron deficiency anemia	Ischemic stroke	7.933	0.160	0.008	0.681	0.214
	Large artery stroke	0.070	0.966	0.0003	0.998	NA
	Cardioembolic stroke	5.600	0.469	−0.018	0.544	0.441
	Small vessel stroke	6.803	0.236	0.034	0.416	0.255
Ischemic stroke	Iron deficiency anemia	3.345	0.764	−0.037	0.369	0.764
Large artery stroke		10.302	0.244	−0.050	0.074	0.274
Cardioembolic stroke		1.321	0.724	0.010	0.650	0.669
Small vessel stroke		3.586	0.465	−0.014	0.708	0.485

NA, not available.

## 4 Discussion

Employing available GWAS datasets and bidirectional two-sample MR methodologies, this research probed the causal connections between IDA and IS, as well as its specific subtypes. The MR analysis was executed through the IVW approach, MR-Egger, and the weighted median estimation technique, accompanied by rigorous sensitivity analyses to mitigate confounding influences and solidify result validity. The results revealed no causal correlation between IDA and any IS, or its LAS, CES, and SVS subtypes. Furthermore, the exploration of reverse causality did not uphold a causal influence of IS and its subtypes on IDA. This study stands as the first effort to elucidate a potential causal relationship between IDA and SVS.

Earlier research has predominantly focused on the link between anemia and IS. These studies, particularly long-term observational follow-ups, have demonstrated that individuals with anemia were at approximately a 1.5 times greater risk of developing IS (32, 33). Further investigation has found that within the span of one to two years, anemia could heighten the risk of IS by 1.6 and 1.35 times, respectively (34). Additionally, individuals with chronic kidney disease who also suffer from anemia have been identified as having a heightened risk for stroke (35). Despite these associations, a recent MR study examining anemia and cardiovascular disease has concluded that anemia did not causally impact IS (36). On this basis, the discoveries from our investigation contribute novel evidence supporting the non-causal associations between IDA and IS, inclusive of its various subtypes.

While the majority of preceding experimental and observational research pointed toward a possible association between IDA and IS, the precise mechanism through which IDA contributes to IS remains partially understood. Several hypotheses have been advanced, positing that IDA may lead to IS through the formation of a hypercoagulable state, a direct consequence of iron deficiency or anemia itself. Secondary thrombocytosis due to IDA and the resultant anemia-induced hypoxia can create a disparity between the supply and demand of oxygen in end-arteries, precipitating ischemia and infarction (15). Anemia is known to cause hyperdynamic circulation, which in turn increases molecular adhesion expression on vascular endothelial cells (37), triggering an inflammatory response that may result in thrombosis, akin to atherosclerotic processes (38). In the context of IDA, there is an increase in erythropoietin secretion, which not only augments red blood cell count but also stimulates platelet production, thereby inducing thrombocytosis and thrombus formation (39). On the cellular level, diminished hemoglobin levels impair oxygen delivery to tissues, potentially leading to hypoxia. Exposure of arteries to hypoxic conditions initiates signaling in monocytes/macrophages and T lymphocytes via endothelial cells, smooth muscle cells, and fibroblasts, which may hasten atheroma development (40, 41). Hypoxia can also disrupt the mitochondrial respiratory chain, severely affecting energy production. Given the high oxygen demands of neurons, they are particularly vulnerable to hypoxic conditions. Research by Akins et al. has indicated that turbulent blood flow, a common occurrence in severe anemia, can damage vascular endothelium and promote platelet aggregation and clot formation (12). Consequently, while our study did not substantiate a direct elevation in IS risk attributable to IDA, the possibility of anemia contributing to thrombosis warrants attention.

Additionally, the potential link between iron deficiency and IS was also a research focus that cannot be ignored. Sequential research following the First National Health and Nutrition Examination Survey (NHANES I) unveiled a pronounced U-shaped correlation between transferrin saturation levels and stroke incidence among Caucasian females aged 45–74 (42), indicating heightened stroke risks at both deficient and excessive iron levels in the bloodstream. Similarly, Ekblom et al. found an elevated risk of stroke within the highest quartile of total iron binding capacity-a metric that escalates with iron deficiency (43). The correlation between iron deficiency and thrombophilia has gained increasing recognition over recent years (44–46). Iron deficiency is linked to a spectrum of thrombotic conditions, including cerebral venous sinus thrombosis (47) and carotid artery thrombosis (12), as well as numerous cases of embolic stroke (48) and IS (14, 49). Epidemiological research also delineated a higher prevalence of IDA among individuals with cerebral venous thrombosis compared to a control group (50). Diverging from earlier research, our bidirectional MR analysis revealed no causal link between IDA and (IS), including its various subtypes. Nonetheless, prior investigations suggested that the combined impact of anemia and iron deficiency may have a higher propensity for promoting blood clot formation (51). Evidence suggests that IDA can precipitate IS through a triad of mechanisms: (i) Reduced hemoglobin levels precipitate a hypoxic condition; (ii) Hypoxia induces transferrin production via hypoxia-inducible factor-1, which then activates and interacts with thrombin/factor XIIa, impairing the activity of anticoagulant proteases, thus leading to a hypercoagulable state; (iii) IDA stimulates thrombin generation by platelets, resulting in thrombocytosis (51). These interconnected mechanisms collectively exacerbate cerebral ischemic damage, elevating the risk of IS in the context of IDA.

A recent two-sample bidirectional MR study has elucidated that IS increases the risk of anemia (36). Our findings further delineate that IS does not augment the risk for IDA, hinting at a potential causative linkage between IS and other anemia subtypes, warranting additional scrutiny. In addition, the relatively extensive 95% CI in our MR-Egger analysis signals instability in the causal effect of IS on IDA, possibly due to the scarce IVs employed in the analysis. Hence, the conclusions drawn regarding the causal effect of IS on IDA necessitate further empirical corroboration upon the refreshment of the GWAS database. Nonetheless, our study is distinguished by several strengths. First, MR analysis sidesteps issues like reverse causation and confounding variables common in observational research, offering a more time-and resource-efficient approach to dissecting complex disease etiologies. Second, the deployment of summary-level data from individuals of European ancestry, encompassing a vast array of IS subtype cases and controls, minimizes the risk of bias from population stratification. Third, leveraging publicly accessible GWAS summary statistics capitalizes on a broad sample size, ensuring more accurate estimates and stronger statistical power, thereby reducing research expenses, enhancing bioinformatics use, and bolstering the credibility of our findings.

Our study inevitably encounters certain limitations. First, the GWAS summary statistics utilized herein are exclusively sourced from European cohorts, casting uncertainty on the generalizability of our conclusions across diverse ethnicities. The necessity for future corroboration of our results with large-scale GWAS summary statistics encompassing varied populations is paramount. Second, the aggregate data used in our analysis restricts access to detailed individual-level information. Third, akin to all MR studies, our study is unable to completely exclude the influence of unobserved pleiotropy, potentially introducing bias into our results. This underscores the need for extensive further research to elucidate the relationship between IDA and IS. Fourth, the study failed to identify a causal effect of IDA on LAS, with the MR-Egger analysis displaying broad 95% CIs, indicating the potential instability of these outcomes and the necessity for further inquiry.

## 5 Conclusion

In summary, our research furnished evidence advocating for no causative influence of IDA on the susceptibility to IS, LAS, CES or SVS. Bidirectional MR analysis yielded no evidence of a causal relationship between IDA and any IS, LAS, CES and SVS. Future studies should employ well-conducted clinical trials, featuring robust methodologies and substantial numbers of participants, to delve deeper into the mechanisms connecting IDA to IS and its various subtypes.

## Data Availability

The original contributions presented in the study are included in the article/Supplementary material, further inquiries can be directed to the corresponding author.

## Footnotes

^1^ https://r10.finngen.fi/

^2^ https://www.ebi.ac.uk/gwas/
