# Supplementary material for: No genetic association between iron deficiency anemia and ischemic stroke and its subtypes: a bidirectional two-sample Mendelian randomization study
Source: Front Neurol. 2024 Aug 19;15:1408758. doi: 10.3389/fneur.2024.1408758 (PMC11369898; doi:10.3389/fneur.2024.1408758)

**FIGURE S1** Forest plots of ischemic stroke and its subtypes on iron deficiency anemia. (A) Ischemic stroke; (B) Large artery stroke; (C) Cardioembolic stroke; (D) Small vessel stroke.


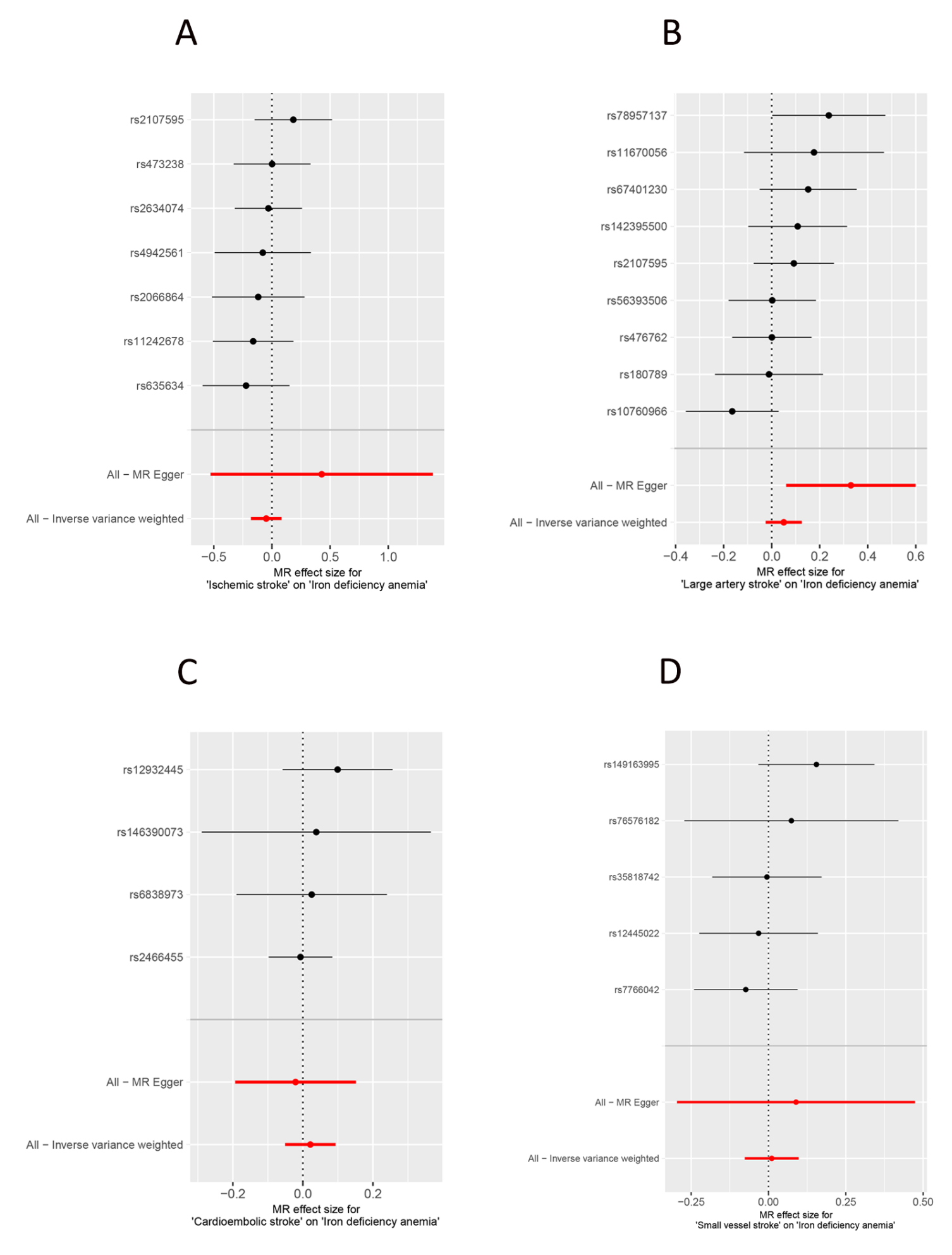


**FIGURE S2** MR analysis scatter plots of ischemic stroke and its subtypes on iron deficiency anemia. (A) Ischemic stroke; (B) Large artery stroke; (C) Cardioembolic stroke; (D) Small vessel stroke.


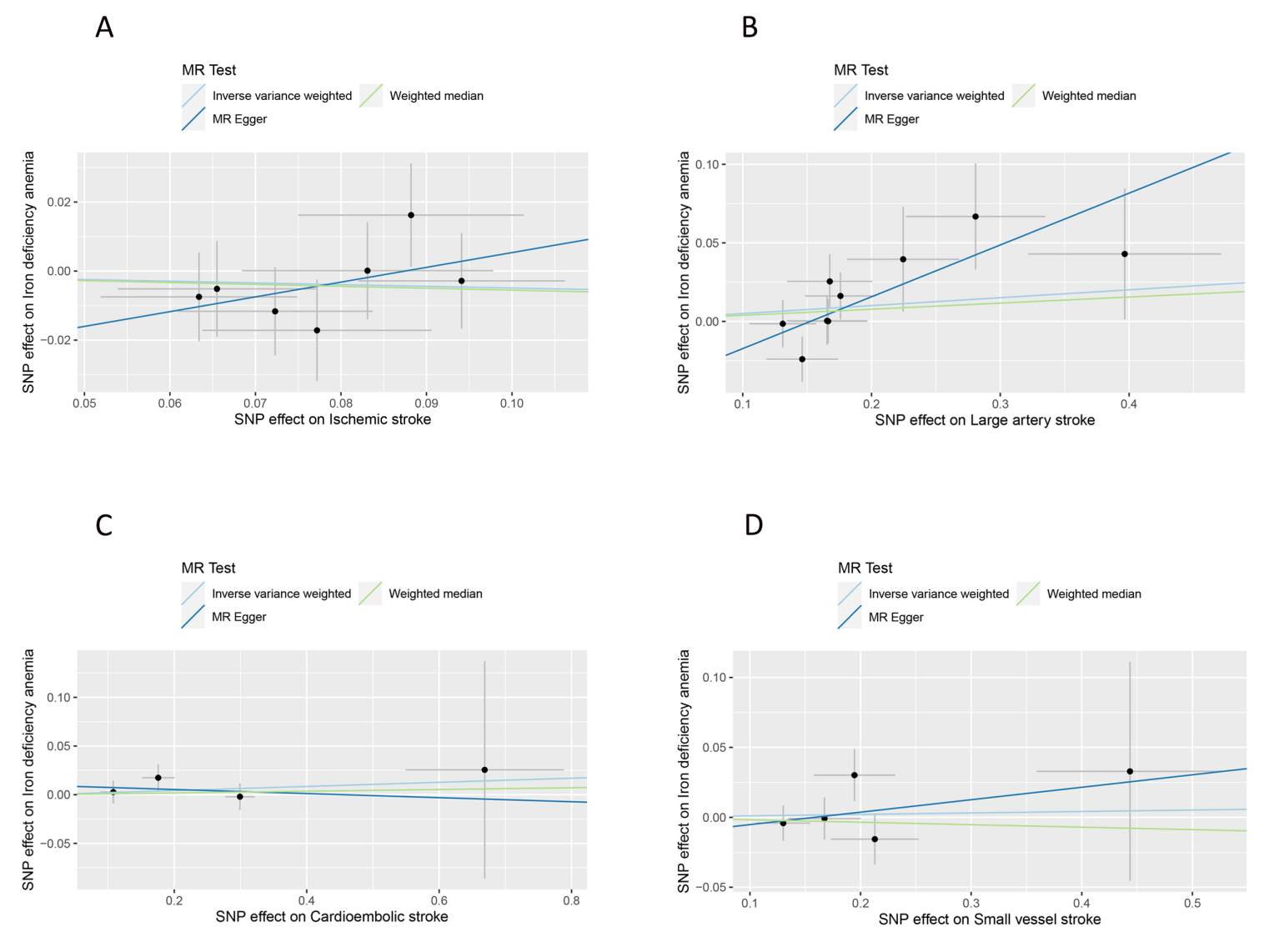


**FIGURE S3** Leave-one-out analysis of iron deficiency anemia on ischemic stroke and its subtypes. (A) Ischemic stroke; (B) Large artery stroke; (C) Cardioembolic stroke; (D) Small vessel stroke.


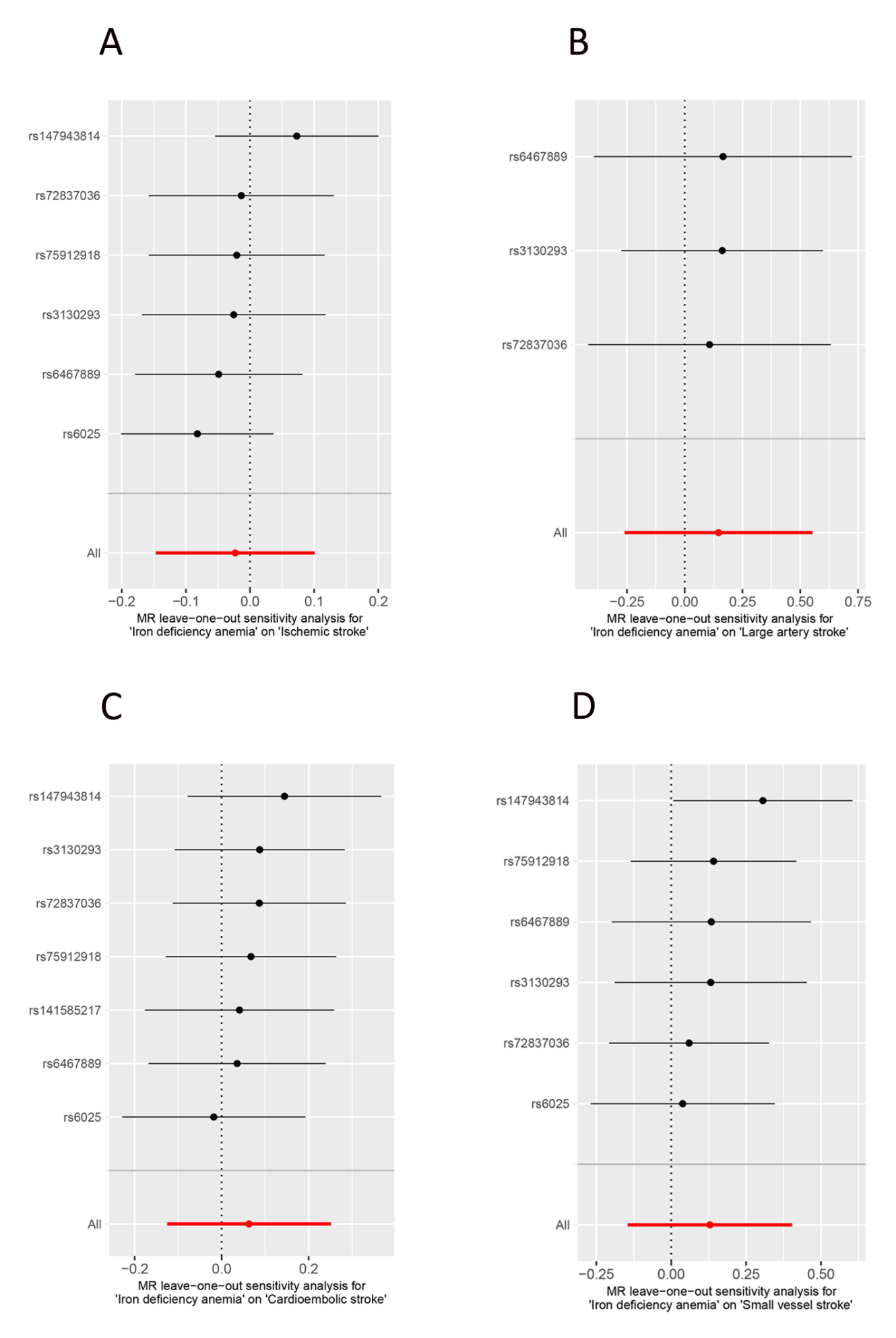


**FIGURE S4** Leave-one-out analysis of ischemic stroke and its subtypes on iron deficiency anemia. (A) Ischemic stroke; (B) Large artery stroke; (C) Cardioembolic stroke; (D) Small vessel stroke.


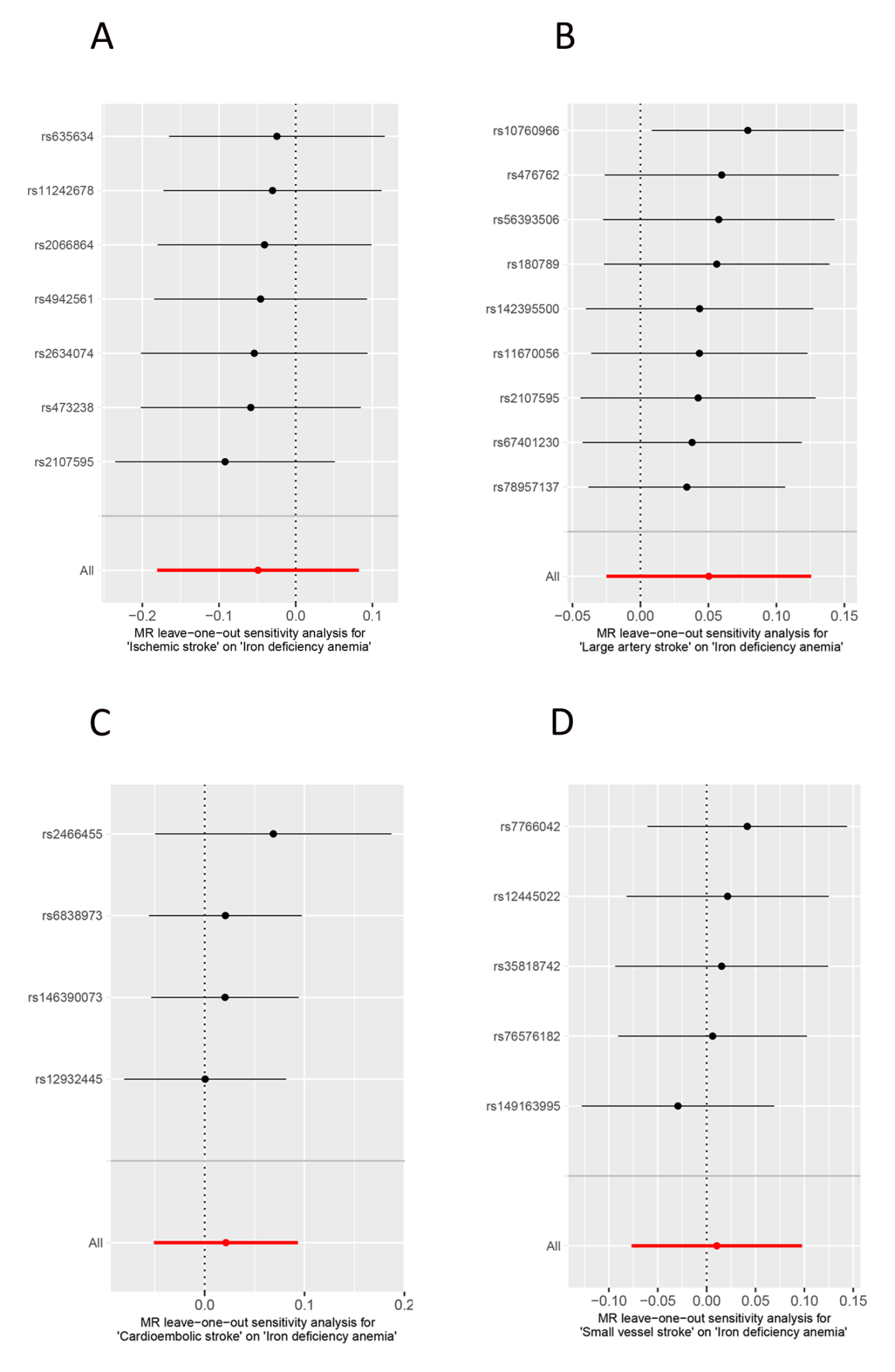


**FIGURE S5** Funnel plots of the results from MR analysis of iron deficiency anemia on ischemic stroke and its subtypes. (A) Ischemic stroke; (B) Large artery stroke; (C) Cardioembolic stroke; (D) Small vessel stroke.


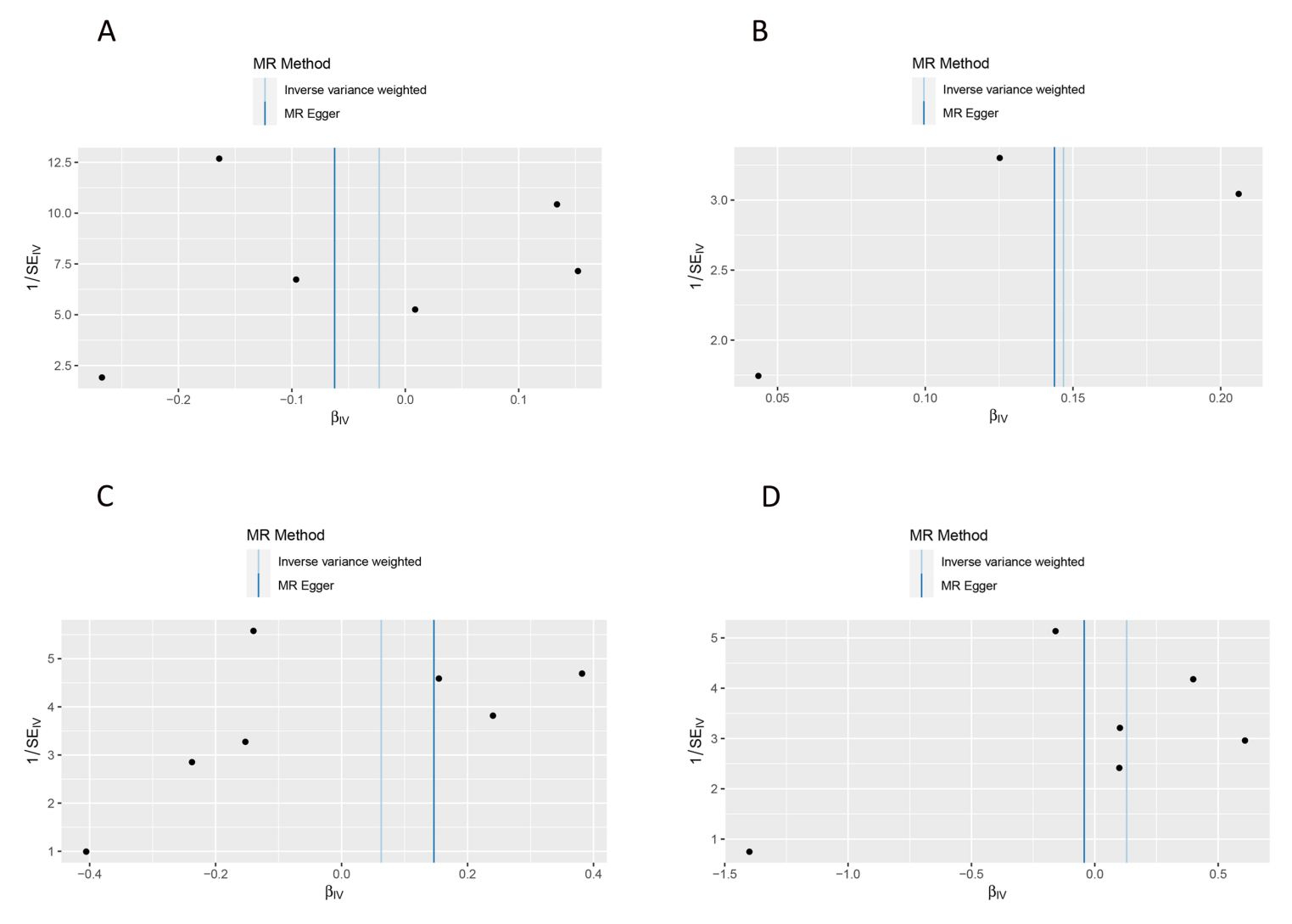


**FIGURE S6** Funnel plots of the results from MR analysis of ischemic stroke and its subtypes on iron deficiency anemia. (A) Ischemic stroke; (B) Large artery stroke; (C) Cardioembolic stroke; (D) Small vessel stroke.


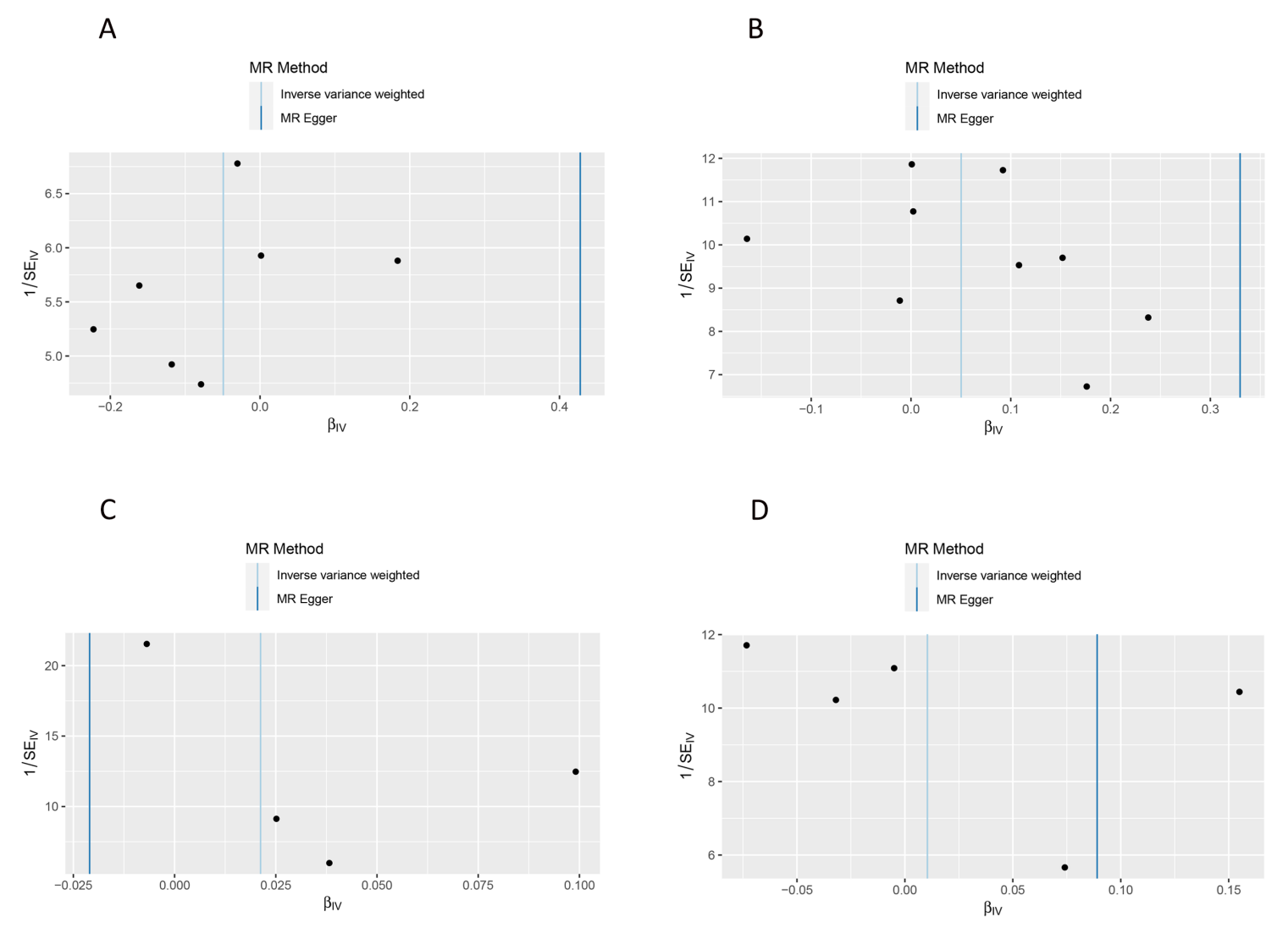

Supplement: Supplementary file 2 [file Data_Sheet_1.DOCX]
